# Alterations of right ventricular assessment by use of an ultrasound-enhancing agent during routine visualization of the left ventricle

**DOI:** 10.1093/ehjimp/qyag004

**Published:** 2026-01-13

**Authors:** Ruchika Bhargav, Raphael Bonita, Alekhya Potluri, Chiduzie Madubata, Nazanin Moghbeli, Hamza Akhtar, Sarah Morris, Bridget Clift, Gregg S Pressman

**Affiliations:** Division of Cardiovascular Diseases and Hypertension, Rutgers Robert Wood Johnson University Hospital, 125 Paterson Street, ET-800, New Brunswick, NJ 08901, USA; Division of Cardiology, Heart and Vascular Institute, Jefferson Einstein Hospital, Philadelphia, PA 19141, USA; Division of Cardiology, Heart and Vascular Institute, Jefferson Einstein Hospital, Philadelphia, PA 19141, USA; Division of Cardiology, Heart and Vascular Institute, Jefferson Einstein Hospital, Philadelphia, PA 19141, USA; Division of Cardiology, Heart and Vascular Institute, Jefferson Einstein Hospital, Philadelphia, PA 19141, USA; Division of Cardiology, Heart and Vascular Institute, Jefferson Einstein Hospital, Philadelphia, PA 19141, USA; Division of Cardiology, Heart and Vascular Institute, Jefferson Einstein Hospital, Philadelphia, PA 19141, USA; Division of Cardiology, Heart and Vascular Institute, Jefferson Einstein Hospital, Philadelphia, PA 19141, USA; Division of Cardiology, Heart and Vascular Institute, Jefferson Einstein Hospital, Philadelphia, PA 19141, USA

**Keywords:** right ventricle assessment, ultrasound enhancing agent, Apical 4-chamber view, right ventricle-focused view

## Abstract

**Aims:**

Two-dimensional echocardiography remains the primary means of right ventricular (RV) evaluation. This study evaluated effects of ultrasound contrast, employed to improve left ventricular (LV) visualization, on assessment of RV size and function.

**Methods and results:**

In 100 consecutive patients where an ultrasound enhancing agent (UEA) was indicated to improve LV visualization, apical 4-chamber (A4C) views were collected before and after contrast injection, maintaining transducer position. For the last 50 subjects, RV-focused (RVF) views were also obtained. Images were presented in random order to four echocardiographers with varying levels of experience. They were tasked with determining which ventricle formed the apex, whether the RV free wall was adequately visualized, assessing RV size and contractility and reporting their confidence level in their readings. In both A4C and RVF views, the LV was less often determined to be apex-forming when UEA was used and RV size assessments moved in the direction of dilated for all readers. Similarly, a determination of reduced RV contractility occurred more often with UEA. Many of these differences were statistically significant. Interestingly, RV free wall visualization was not improved with contrast use. Moreover, reader confidence in interpretation was actually reduced when contrast was employed, with statistically significant differences for all four readers in the A4C view and two readers in the RVF view.

**Conclusion:**

Opportunistic evaluation of the RV after employing UEA resulted in significant differences in visual assessment of various RV parameters. Reader confidence in interpretation was not improved when contrast was used.

## Graphical Abstract

**Graphical Abstract:**
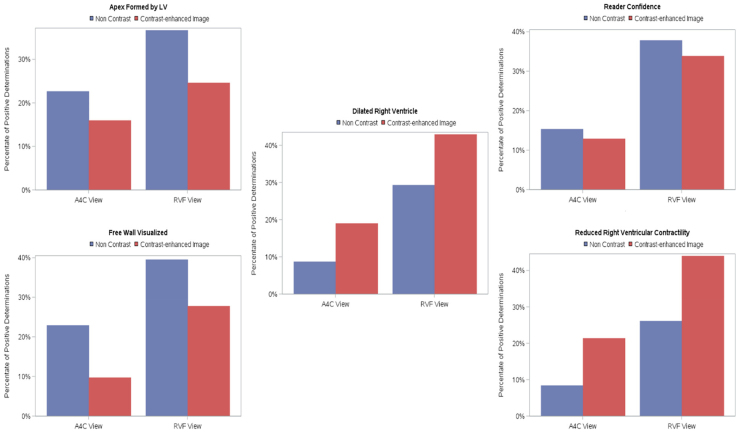
The presence of UEA, injected according to a LV opacification protocol, can alter reader perception of right ventricular assessment in both the A4C and RVF views. When UEA is utilized, the RV can appear to be apex-forming or apex-sharing, can appear larger and less contractile and the RV free wall definition is not improved on contrast-enhanced images. Interestingly, reader confidence in assessing the RV is not improved by use of UEA. A4C, apical-4-chamber; LV, left ventricle; RV, right ventricle; RVF, right ventricle-focused; UEA, ultrasound-enhancing agent.

## Introduction

There is increasing interest in the right ventricle (RV) due to its prognostic significance in pulmonary embolism, pulmonary hypertension, valvular heart disease, ischaemic heart disease, and heart failure.^1–4^ Accurate assessment of RV size and function is thus important for appropriate risk stratification of patients. Despite increasing use of advanced imaging techniques [e.g. three-dimensional echocardiography, cardiac magnetic resonance (CMR), and cardiac computed tomography], two-dimensional echocardiography (2DE) remains the primary imaging modality for the RV.^5^

As per the 2025 American Society of Echocardiography guidelines for echocardiographic assessment of the right heart in adults and special considerations in pulmonary hypertension, assessment of the right ventricle should include both qualitative and quantitative parameters: fractional area change, Doppler Tissue Imaging derived tricuspid lateral annular systolic velocity wave (S’), tricuspid annular plane systolic excursion (TAPSE), tissue motion annular displacement (TMAD), 3-dimensional right ventricular ejection fraction (3D RVEF), and RV myocardial performance index (Tei index). Quantitative measures are preferred, and the normal values are based on published mean and standard deviation data, which is obtained from adult individuals without history of heart and pulmonary disease.^6^ However, right ventricular assessment by 2DE is challenging because of the right ventricle’s retrosternal location, complex geometric shape, extensive trabeculations, and unique contraction pattern.^7^ Therefore, CMR remains the gold standard for the evaluation of RV size and function. Several studies have compared 2DE against CMR in the assessment of RV but no single 2DE parameter has proven to consistently correlate with CMR derived RV size and systolic function.^8^

Ultrasound-enhancing agents (UEA) are recommended when two or more left ventricular (LV) segments are not optimally visualized.^9^ A few studies suggest that echocardiographic contrast may allow more accurate assessment of RV wall motion and function on 2DE.^10,11^ We hypothesized that the use of microbubble contrast injections to optimize visualization of the left ventricle would also benefit visualization of the right ventricle and alter reader assessment of right ventricular size and function. Given that contrast penetrates to the compact myocardium, we further hypothesized that the RV would appear larger on contrast-enhanced images and more often apex-forming. Finally, we hypothesized that use of UEA would increase reader confidence when interpreting RV contractility.

## Methods

From March 2022, patients who had clinically indicated echocardiograms requiring the use of UEA (perflutren lipid microsphere, Definity®), for LV definition, were identified at the time echocardiography was performed. Those in whom the contrast injection was suboptimal were excluded. Subjects were otherwise unselected and were enrolled until we reached a total set of 100 studies. All studies were performed by one of two sonographers, experienced in contrast administration, according to our laboratory’s protocol. Various ultrasound machines were used by the sonographers: Philips Affinity and Epiq, GE S70 and e95. The study was approved by the Institutional Review Board of Jefferson Einstein Hospital.

2DE was performed according to our institution’s standard protocol, in accordance with ASE guidelines.^12^ Prior to administration of UEA, the sonographer acquired an apical four-chamber view (A4C) following which Definity® was injected. Without changing probe position, a second identical A4C image was acquired after contrast filled the right ventricle and then adequately visualized the left ventricle. The rest of the images were collected in usual fashion. The standard contrast settings included frequency of 24 Hz, mechanical index of 0.19, and overall gain at 50%; these settings were adjusted based on image quality and patient’s body habitus. About 1.5 mL of Definity was diluted with 8.5 mL of normal saline, and a 2 mL bolus was injected over 20 s duration (at a rate of 1 mL per 10 s). A repeat bolus of 1–2 mL could have been provided at the discretion of the sonographer if the image quality was suboptimal. Midway through the study, we added collection of pre- and post-contrast RV-focused (RVF) images (50 subjects) as well. Thus, in total, 100 subjects were included, all of whom had non-contrast and contrast-enhanced A4C views; the final 50 subjects also had non-contrast and contrast-enhanced RVF views. Right ventricular free wall strain was not routinely performed during the acquisition of the studies.

Four readers were picked from our faculty to review the studies in a blinded manner. All were board certified in echocardiography but had varying levels of experience: two junior readers had < 10 years’ experience while two senior readers had >15 years’ experience. Each reader reviewed all 300 clips (100 each of contrast and non-contrast A4C views, and 50 each of contrast and non-contrast RVF views). Each clip had personal identifiers removed and was assigned a unique serial number using an online random number generator. The clips were then presented to the readers in random order. Readers were required to evaluate the following: (i) which ventricle was apex-forming (LV, RV, or both), (ii) RV size (normal, mildly enlarged, moderately enlarged, severely enlarged), (iii) RV contractility (normal, mildly reduced, moderately reduced, severely reduced), and (iv) adequate RV free wall visualization (yes or no). We also asked readers to state their confidence in their overall assessment of the RV (very confident, somewhat confident, not very confident, not at all confident). The readers were not given additional instructions and no criteria were defined in determining adequacy of RV free wall visualization and ratings of confidence level. Quantitative measures were not performed by the readers and evaluation of all the RV parameters was based solely on visual assessment. Finally, readers were informally queried on what factors affected their confidence level.

### Statistical methods

To make statistical comparisons simpler and more easily understood, those with abnormal values were combined and compared as a group to those with normal findings. Specifically, for RV size and contractility, the number with ‘normal’ findings was compared to the number with ‘abnormal findings’ (mild, moderate, or severe). For apex-forming, the number of LV-forming subjects was compared to the combined number of RV-forming or shared-apex subjects. For reader confidence, ‘confident’ was compared to all degrees of non-confidence (somewhat confident, not very confident, not at all confident). RV free wall visualization was compared as collected, either yes or no.

For each reader, comparisons were made between non-contrast and contrast-enhanced A4C and RVF views for each parameter. Each of the above variables was assessed using chi-square analysis with a continuity correction for each outcome and each reader.

## Results

Mean age of the 100 subjects was 59.4 ± 15.1 years; 57% were male, 52% were outpatients, and 69% were African American (*Table 1*).

**Table 1:** Demographics and echocardiogram characteristics

Characteristic	Patients
Age (years)	59.4 ± 15.1
Body mass index (kg/m²)	30.9 ± 7.6
Gender	
Male	57 (57)
Female	43 (43)
Race and ethnic group	
African-American	69 (69)
White	4 (4)
Hispanic	6 (6)
Asian	3 (3)
Native American	6 (6)
Unknown	12 (12)
Outpatients	52 (52)
Inpatients	48 (48)
Left ventricle size*	
Normal	82 (82)
Dilated	18 (18)
Right ventricle size*	
Normal	82 (82)
Dilated	18 (18)
Left ventricular ejection fraction*	
>50%	59 (59)
40–50%	13 (13)
30–40%	8 (8)
<30%	20 (20)
Right ventricular systolic function*	
Normal	84 (84)
Reduced contractility	16 (16)

^*^As stated on the official echocardiogram report.

Data are expressed as mean ± standard deviation or as number (percentage).

### Study parameter: which ventricle formed the apex

**Figure 1:**
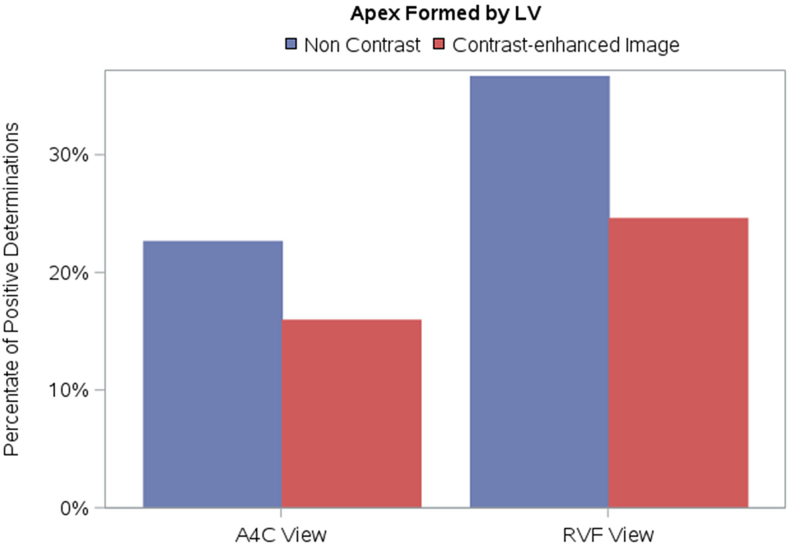
Impact of UEA on determination of which ventricle forms the apex in A4C and RVF views. There is a shift towards RV or both ventricles forming the apex as opposed to LV forming the apex on contrast-enhanced images in both A4C and RVF views.

**Figure 2:**
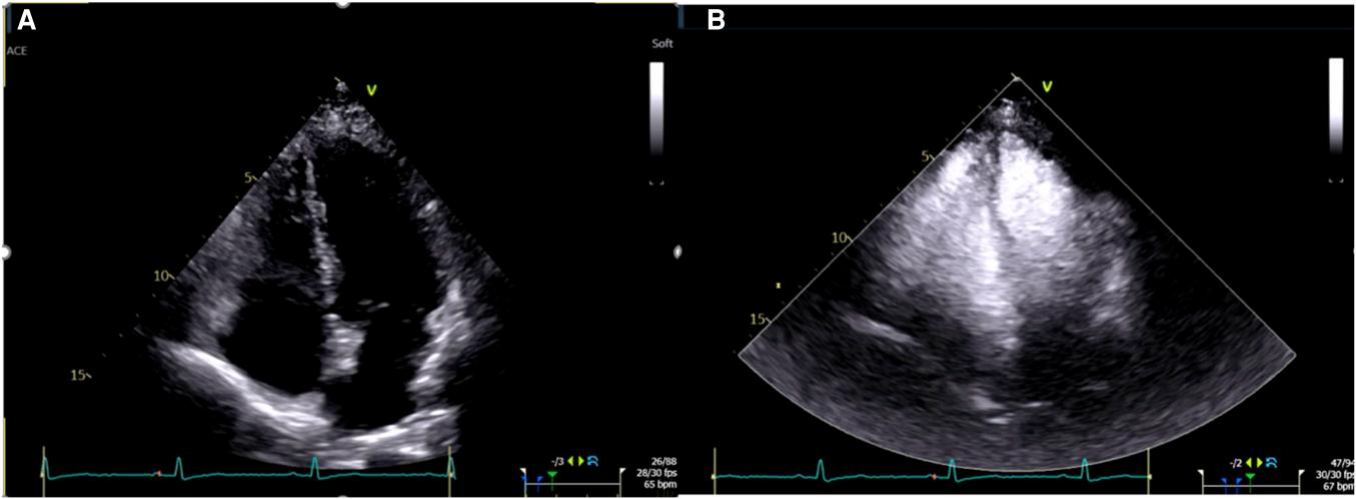
Non-contrast apical four-chamber view (*A*) and contrast-enhanced apical four-chamber view (*B*) on transthoracic echocardiogram.

Regarding which ventricle formed the apex (LV, RV, or both), there were notable differences of interpretation when comparing non-contrast with contrast-enhanced images. *Figure 1* reveals the trend of the shift towards RV or both ventricles forming the apex as opposed to LV forming the apex on contrast-enhanced images in both A4C and RVF views. For the A4C view, there was a shift from the LV forming the apex to other choices (such as RV forming the apex or both ventricles sharing the apex) for all readers; these differences were statistically significant for 3 readers (*Table 2*). As an example, *Figure 2* depicts a non-contrast A4C view, which was interpreted by all four readers as showing the LV forming the apex, and a contrast-enhanced A4C view of the same patient, which was judged by three out of four readers as the RV to be apex forming or apex sharing. The shift away from the LV forming the apex was also observed for all four readers in the RVF view, though it was statistically significant for only two readers (*Table 3*).

**Table 2:** Apical four-chamber view results (*n* = 100)

Reader		Apex Formed by LV		RV dilated		RV contractility reduced		Free wall visualized		Confident	
	Contrast	Yes	No	No	Yes	No	Yes	Yes	No	Yes	No
1	No	49	51	61*	39	86**	14	63**	37	73**	27
	Yes	38	62	43	57	50	50	18	82	35	65
2	No	67**	33	60**	40	66**	34	79**	21	48**	52
	Yes	42	58	34	66	35	65	48	52	28	72
3	No	70**	30	55**	45	60**	40	49*	51	35**	65
	Yes	46	54	22	78	21	79	31	69	13	87
4	No	72*	28	72**	28	75**	25	72*	28	67**	33
	Yes	54	46	51	49	52	48	46	54	43	57

^*^P-value <0.05.

^**^P-value <0.01.

**Table 3:** Right ventricle–focused view results (*n* = 50)

Reader		Apex Formed by LV		RV dilated		RV contractility reduced		Free wall visualized		Confident	
	Contrast	Yes	No	No	Yes	No	Yes	Yes	No	Yes	No
1	No	23*	27	26	24	36**	14	27**	23	38**	12
	Yes	13	37	19	31	19	31	9	41	22	28
2	No	28	22	25*	25	24	26	38	12	16	34
	Yes	20	30	12	38	20	30	37	13	19	31
3	No	29*	21	25**	25	24**	26	32*	18	1	49
	Yes	17	33	7	43	8	42	22	28	1	49
4	No	26	24	36**	14	32*	18	38*	12	26**	24
	Yes	19	31	21	29	22	28	28	22	9	41

^*^P-value <0.05.

^**^P-value <0.01.

### Study parameter: RV size

**Figure 3:**
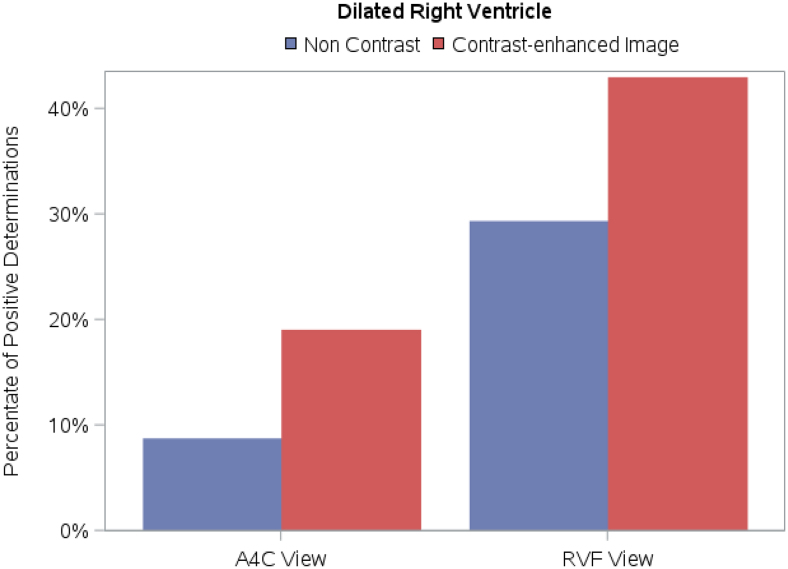
Impact of UEA on determination of RV size in A4C and RVF views. With the use of UEA, there was a shift towards a determination of RV dilation in both the A4C and RVF views.

The four blinded readers were required to grade RV size (normal, mildly enlarged, moderately enlarged, and severely enlarged). With the use of UEA, there was a shift towards a determination of RV dilation (mild, moderate, or severely enlarged) in both the A4C and RVF views, as highlighted in *Figure 3*. Differences were statistically significant for all four readers in the A4C view (*Table 2*) and for three of four readers in the RVF view (*Table 3*). *Video 1* shows non-contrast and contrast-enhanced A4C views of the same patient; two readers judged the RV to be of normal size in the non-contrast image and dilated in the contrast-enhanced image while two readers judged the RV to be mildly dilated in the unenhanced image and at least moderately dilated in the contrast enhanced image.

### Study parameter: RV contractility

**Figure 4:**
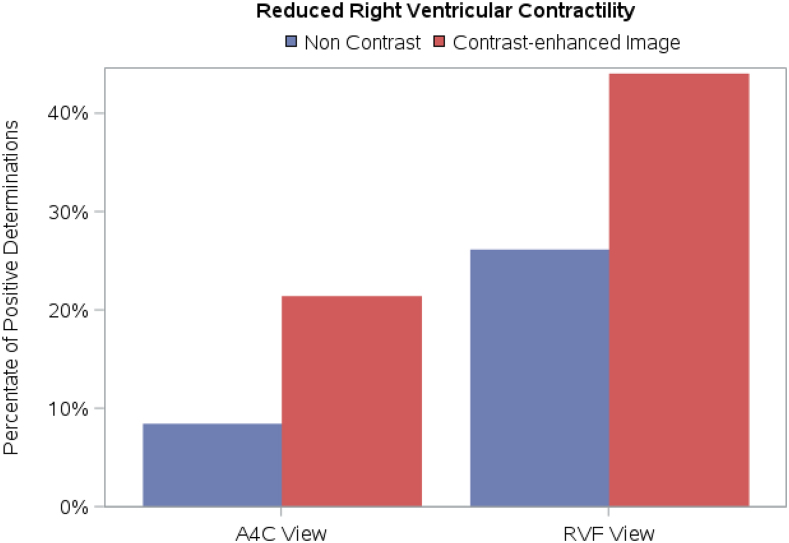
Impact of UEA on determination of RV contractility in A4C and RVF views. For both views, contrast administration led to a shift towards a determination of impaired RV contractility.

The four blinded readers were required to grade RV contractility (normal, mildly reduced, moderately reduced, and severely reduced). For all readers, there were statistically significant differences between contrast and non-contrast images when determining RV contractility in the A4C view (*Table 2*); in the RVF view, there were statistically significant differences for 3 out of 4 readers (*Table 3*). For both views, contrast administration led to a shift towards a determination of impaired RV contractility (*Figure 4*). *Video 1* shows non-contrast and contrast-enhanced A4C views of the same patient; three out of four readers judged the RV to be hypocontractile with contrast use and of normal function in the non-contrast image while one reader judged the RV to be mildly hypocontractile in the unenhanced image and moderately hypocontractile in the contrast enhanced image.

### Study parameter: RV free wall visualization

**Figure 5:**
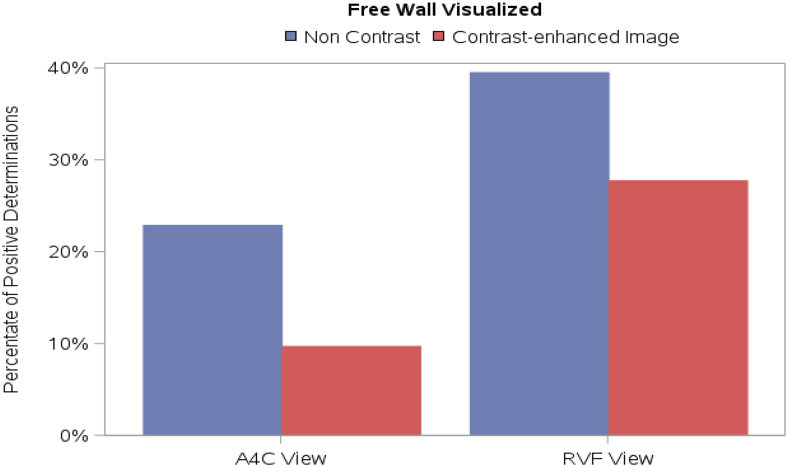
Impact of UEA on RV free wall visualization in A4C and RVF views. With contrast administration, the RV free wall was felt to be less well visualized in both the A4C and RVF views as compared to the non-contrast images.

The four blinded readers were required to specify if they were able to visualize the RV free wall adequately. There were statistically significant differences of interpretation for each reader when comparing non-contrast with contrast images in the A4C view, and for a majority of the readers in the RVF view. Contrary to expectations, with contrast administration the RV free wall was felt to be less well visualized in both the A4C and RVF views as compared to the non-contrast images (*Figure 5*). *Video 2* depicts non-contrast and contrast-enhanced A4C views of the same patient; three out of four readers determined the RV free wall to be inadequately visualized on the contrast images. *Video 3* depicts non-contrast and contrast-enhanced RVF views of the same patient; three out of four readers determined the RV free wall to be inadequately visualized on the contrast images.

### Reader confidence level

**Figure 6:**
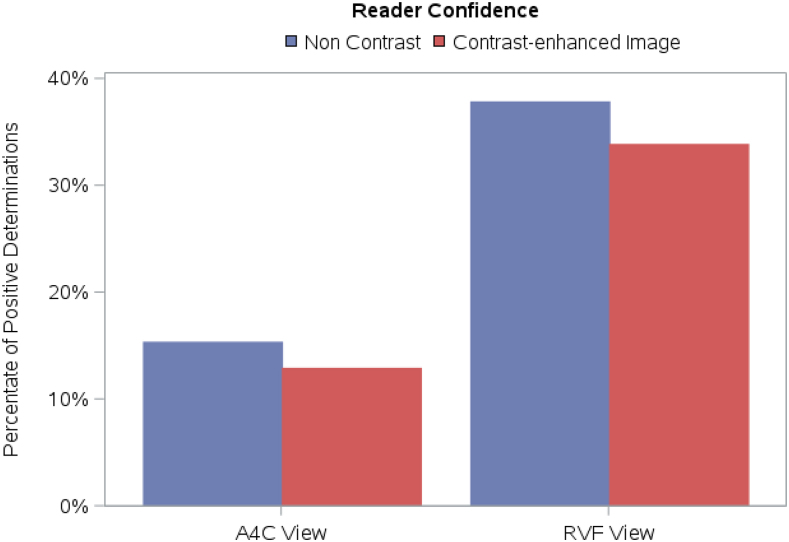
Impact of UEA on reader confidence in RV assessment in A4C and RVF views. Confidence levels did not improve when UEA was utilized in both views.

The four blinded readers were required to grade their confidence level pertaining to overall RV assessment (very confident, somewhat confident, not very confident, not at all confident). There were statistically significant differences in reader confidence for all four readers when comparing contrast with non-contrast images in the A4C view. Contrary to our hypothesis, confidence levels did not improve when UEA was utilized (*Table 2*, *Figure 6*). The drop in confidence was less pronounced in the RVF view but was still significant for two readers (*Table 3*, *Figure 6*). *Video 2* depicts non-contrast and contrast-enhanced A4C views in the same patient; confidence levels did not improve for all four readers during assessment of the contrast-enhanced image.

## Discussion

The main finding of this study is that the presence of UEA, injected according to a LV opacification protocol, alters reader assessment of the RV in important ways: (i) the RV more frequently appears to be apex-forming or apex-sharing when contrast is used, (ii) the RV often appears larger and less contractile on contrast imaging, (iii) RV free wall definition is not improved on contrast-enhanced images, and (iv) reader confidence in assessing the RV is not improved by use of UEA.

The use of UEA improves accuracy of left ventricular wall motion and systolic function assessment but based on the current findings, it appears to produce more variability in RV interpretation. To our knowledge, this is the first study to compare various RV parameters on A4C and RVF views pre- and post-contrast in a blinded, randomized manner. Our results are somewhat surprising and require explanation.

Regarding RV size, we note that contrast penetrates to the compacted myocardium whereas the apparent endocardial border on unenhanced images is mostly due to the trabeculae. As a result, the RV can appear apex-forming/sharing and larger after contrast injection due to contrast filling the interstices between the trabeculae. Contrast imaging has been noted to result in increased left ventricular dimensions and volume measurements^13^; similar effects on RV measurements were anticipated. Given the highly trabeculated nature of the RV, differences in measurements between non-contrast and contrast-enhanced images may be greater than for the LV. This finding is clinically important as many echocardiographers judge RV enlargement by whether or not the RV is apex-forming.

As to assessment of right ventricular contractility, we note that the trabeculae contract with the rest of the ventricle. In animal models, it has been shown that force generation by trabecular myocytes is similar to that of cells from the compact myocardium^14^; it has also been observed that right ventricular trabeculae contract with higher velocity than do left ventricular trabeculae.^15^ Trabecular movement likely participates in reader perception of contractility. Contrast images, by reducing the visual impact of trabecular motion and focusing on motion of the compact myocardium, might thereby give an impression of reduced RV contractility as compared to unenhanced images.

The finding of reduced definition of the RV free wall was unexpected. A possible explanation involves the complex three-dimensional structure of the right ventricle, which wraps around the LV and is pyramidal in shape.^16^ Other factors that might make the RV border appear less distinct include the highly curved nature of the RV free wall, its heavy trabeculation, the thinness of the free wall and its retrosternal location.

It is also noteworthy that the contrast imaging protocol used in this study is designed to improve visualization of the LV. By the time the LV is optimally visualized the concentration of microbubbles in the RV is decreasing, resulting in less crisp definition of the RV endocardial border. In addition, a high concentration of contrast at the LV apex can cause attenuation artifacts that limit visualization of the basal segments of the ventricle^17^; this might affect RV visualization as well.

Interestingly, we observed that use of contrast affected RV interpretation in the RVF view as well as the A4C view. It is recognized that the A4C view is suboptimal for assessment of RV size and function. The RVF view avoids RV foreshortening and better displays the largest basal diameter and long axis dimension of the RV. It is obtained through lateral displacement of the transducer from the conventional A4C position together with progressive probe rotation.^18^ It appears, however, that introduction of UEA affects reader interpretation of RV size and function in this view as well.

Our study has certain limitations. Only patients deemed to need echocardiographic contrast for LV visualization were included and not a general echocardiography population. However, we did find that opportunistic evaluation of the RV, when contrast is used to enhance LV visualization, can lead to uncertainty as to RV size and function on visual assessment. In addition, multiple echocardiography machines were used, and it is possible that the difficulties of interpretation did not extend equally to each machine or vendor. Also, the echocardiograms were performed in the course of daily clinical practice and acquired in a high-volume lab; such studies are not comparable to the highly detailed studies done specifically for research purposes. However, our findings do have relevance to ‘real world’ practice. Most importantly, our study does not include quantitative assessment of the RV and the findings were not compared to the gold standard, CMR. Rather, our aim was to investigate how interpretation of RV size and function changed after contrast injection, similar to what occurs in many echocardiographic laboratories throughout the world. It will be important for future studies to determine whether use of RV-specific UEA protocols can improve accuracy or assessment as compared with CMR. For this, quantitative studies investigating differences in RV parameters between contrast-enhanced and non-contrast images would be helpful.^9^

In conclusion, the presence of UEA, injected according to a LV opacification protocol, can alter reader perception of right ventricular assessment. This pertains to both the A4C and RVF views. Some of the discrepancies revealed in this study may be due to the complex shape of the RV as well as the use of a left ventricular imaging protocol. Based on these findings, caution should be used in interpreting RV size and function in the presence of echocardiographic contrast until a RV-specific opacification protocol is established and validated.

## Data Availability

Data available on request.

## Lead author biography

Dr. Ruchika Bhargav is a non-invasive cardiologist working at Rutgers Robert Wood Johnson University Hospital in New Jersey, USA. She is a Fellow of the American College of Cardiology as well as a member of the American Heart Association.
